# *Leptanilla hypodracos* sp. n., a new species of the cryptic ant genus *Leptanilla* (Hymenoptera, Formicidae) from Singapore, with new distribution data and an updated key to Oriental *Leptanilla* species

**DOI:** 10.3897/zookeys.551.6686

**Published:** 2016-01-11

**Authors:** Mark K. L. Wong, Benoit Guénard

**Affiliations:** 1 National Parks Board, Singapore Botanic Gardens, 1 Cluny Road, Singapore; 2 School of Biological Sciences, The University of Hong Kong, Pokfulam Road, Hong Kong

**Keywords:** Leptanilla, Leptanillinae, Singapore, Hong Kong, Asia, hypogaeic

## Abstract

A new species of the cryptic and rarely collected ant genus *Leptanilla* is described. *Leptanilla hypodracos* **sp. n.** is the first *Leptanilla* recorded from Singapore in over a century since *Leptanilla havilandi* Forel, 1901 and represents the fourth species of *Leptanilla* known from the Malay Peninsula. An updated key to the *Leptanilla* of the Oriental region is presented. Taxonomic comparisons between *Leptanilla hypodracos* **sp. n.** and four morphologically similar species are provided with particular attention given to *Leptanilla clypeata* Yamane & Ito, 2001, for which new measurements and indices are presented. The first report is presented for the Leptanillinae subfamily from the southeastern part of China with a worker of the genus *Leptanilla* collected in Hong Kong. Finally, the potential of subterranean bait to collect *Leptanilla* species is discussed.

## Introduction

Leptanillines are among the most rarely collected of ant subfamilies due to their minute size and hypogaeic life habits. Although Leptanillinae are widely distributed throughout the Old World and Australian regions, records of the various taxa are remarkably patchy (Antmaps 2015). This is most likely an artefact of a shortage of sampling efforts specifically targeting hypogaeic ants in many regions.

The genus *Leptanilla* Emery, 1870 includes 45 valid species (Bolton 2015). Little information is available about the biology of *Leptanilla* species, although they are believed to be specialist predators of geophilomorph centipedes based on observations of captive colonies of *Leptanilla japonica* Baroni Urbani, 1977 (Masuko 1990) as well as field observations of *Leptanilla taiwanensis* Ogata, Terayama & Masuko, 1995 (Ogata et al. 1995). Masuko (2007) also suggests that the unusually minute cranium or ‘stenocephaly’ in *Leptanilla* larva is a morphological adaptation to facilitate simultaneous group feeding of many individuals on the bodies of their specialized prey. Based on current global distribution records, the Mediterranean region supports the highest diversity of *Leptanilla* species with a total of 17 species and also contains by far the most species-rich countries, Tunisia (seven native species) and Spain (six native species) (Antmaps 2015). The Oriental region follows with a total of 16 described species (Bharti and Kumar 2012; Antmaps 2015) and six more species have been described in the East Palearctic region, all from Japan. However, it is possible that the known diversity of *Leptanilla* species can yet be increased significantly, since these ants have likely been undersampled owing to methodological limitations for effectively collecting small subterranean ants. For example, many *Leptanilla* species have only been described from unassociated males collected in sweep nets and malaise traps (Ogata et al. 1995), while the method proposed for effective collection of subterranean individuals – a combination of the *lavage de terre* and Burlese-Tullgren extraction with large amounts (40kg) of soil (Lopez et al. 1994) – is relatively tedious and time-consuming.

In the Malay Peninsula, three species of *Leptanilla* have been described, and all from the worker caste. *Leptanilla havilandi* Forel, 1901 was described from Singapore, while *Leptanilla butteli* Forel, 1913 was described from Selangor (Malaysia), and *Leptanilla thai* Baroni Urbani, 1977 was described from Khao Chong in southern Thailand. In this paper we describe the worker caste of a new species, *Leptanilla hypodracos* sp. n. from Singapore, which is the fourth *Leptanilla* species from the Malay Peninsula. We also report on a *Leptanilla* worker collected from Hong Kong, which represents the first record of the Leptanillinae subfamily in southeastern China. An updated key to the *Leptanilla* species of the Oriental region (Bharti and Kumar 2012) is presented. Finally, the potential of baited subterranean pitfall traps as a method for collecting cryptic hypogaeic ant taxa such as *Leptanilla* is discussed.

## Methods

Photographs of specimens were obtained with an incorporated digital camera mounted on a Leica M205C dissecting microscope through the Leica Application Suite V4 software. A total of 24 to 86 images were taken and stacked together. Measurements of specimens were taken in mm (accurate to 0.001mm and rounded to the nearest 0.01mm for presentation) with the *Measure Tools* function of the Leica Application Suite V4 software on imaged specimens after proper placement for each body part measured.

The abbreviations used for the measurements and indices are as follows:

HW  Head Width. Maximum width of head in full-face view excluding the eyes.

HL
 Head Length. Maximum length of head from the anterior median clypeal margin to the median posterior margin of the cephalic capsule measured along the midline as a straight line.

MaL
 Mandible Length. Maximum length of mandible from the anterolateral margin of clypeus at outer side of mandibular insertion to mandibular apex.

SL
 Scape Length. Maximum measurable length of scape, from the proximal point of scape shaft, not including the condyle, to the distal end of scape.

EL
 Eye Length. Maximum diameter of eye measured in lateral view.

TL
 Total Length. Maximum length of specimen measured from the tip of the mandibles to the tip of the last abdominal segment, not including sting. Due to the position of the specimen, total length was measured as the sum of head length, mesosoma length, petiole and postpetiole length and gaster length.

WL
 Weber’s Length of Mesosoma. Maximum diagonal distance in lateral view, from base of anterior slope of pronotum to metapleural lobe.

PNW
 Pronotal Width. Maximum width of pronotum measured in dorsal view.

PNH
 Pronotal Height. Maximum height of pronotum measured in dorsal view.

MW
 Mesonotal Width. Maximum width of the mesonotum measured in dorsal view.

PTL
 Petiole Length. In dorsal view, maximum length of petiole, along the sagittal plane, and excluding the peduncle (after Baroni Urbani 1977).

PTH
 Petiole Height. Maximum height of petiole, measured in lateral view from the highest (median) point of the node, orthogonally to the ventral outline of the node (after Baroni Urbani 1977).

PTW
 Petiole Width. Maximum width of the petiole in dorsal view.

PPL
 Postpetiole Length. Maximum length of postpetiole, measured in dorsal view and not excluding the peduncle (after Baroni Urbani 1977).

PPH
 Postpetiole Height. Maximum height of postpetiole, measured in lateral view from the highest point of the node.

PPW
 Postpetiole Width. Maximum width of the postpetiole in dorsal view.

CI
 Cephalic Index. Calculated as: HW / HL × 100.

SI
 Scape Index. Calculated as: SL / HW × 100.

MaI
 Mandibular Index. Calculated as: MaL / HW × 100.

PI
 Petiolar Index. Calculated as: PTW / PTL × 100 (after Baroni Urbani 1977).

PPI
 Postpetiolar Index. Calculated as: PPW / PPL × 100 (after Baroni Urbani 1977).

PPHI
 Postpetiolar Height Index. Calculated as: PPW / PPH × 100 (modified after Bharti and Kumar 2012).

Abbreviations of the type depositories and others are as follows:

LKCNHM
 Lee Kong Chian Natural History Museum, Singapore.

SBSHKU
 Insect Biodiversity and Biogeography Laboratory, School of Biological Sciences, The University of Hong Kong, Hong Kong SAR.

## Results

### Description of new species

#### Leptanilla hypodracos

Taxon classificationAnimaliaHymenopteraFormicidae

Wong & Guénard sp. n.

http://zoobank.org/66F9C713-04C1-40F9-B746-30BD1949AC80

**Figure 1.**
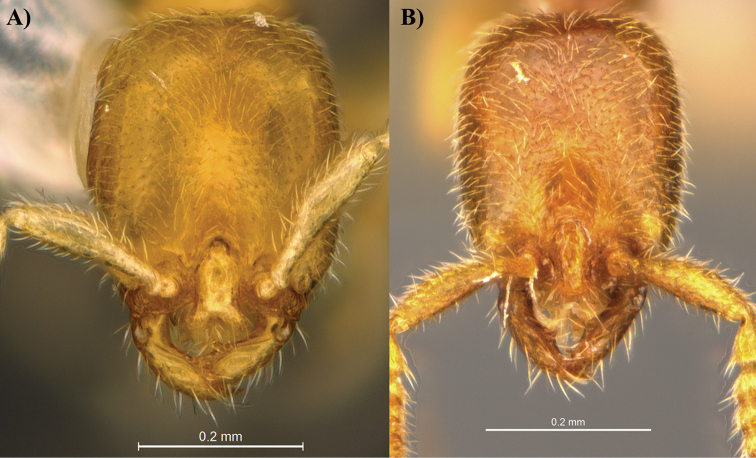
Head view of *Leptanilla clypeata* from Java (**A**) and *Leptanilla hypodracos* from Singapore (Holotype) (**B**). Photographs are on the same scale.

**Figure 2.**
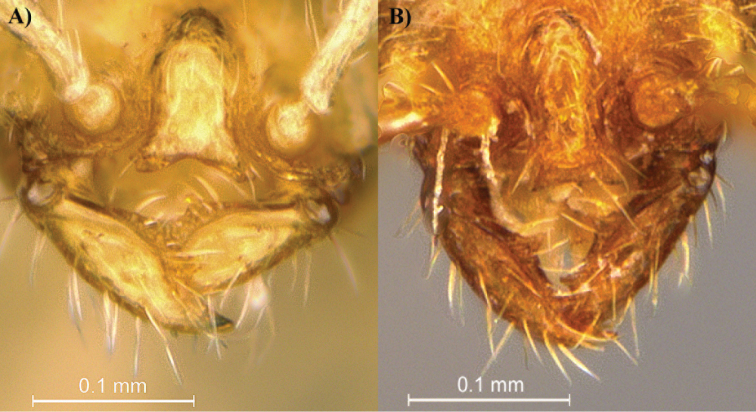
Head view focusing on the mandibles of *Leptanilla clypeata* from Java (**A**) and *Leptanilla hypodracos* from Singapore (Holotype) (**B**). Photographs are on the same scale.

**Figure 3.**
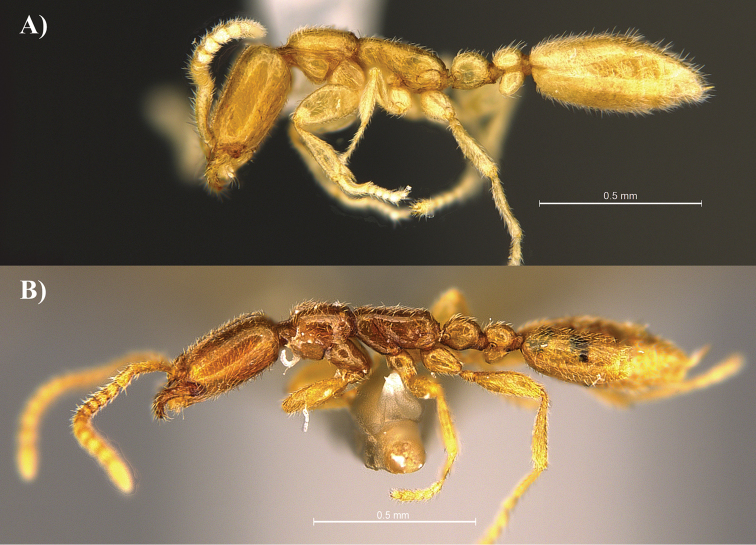
Profile view of *Leptanilla clypeata* from Java (**A**) and *Leptanilla hypodracos* from Singapore (Holotype (**B**). Photographs are on the same scale.

**Figure 4.**
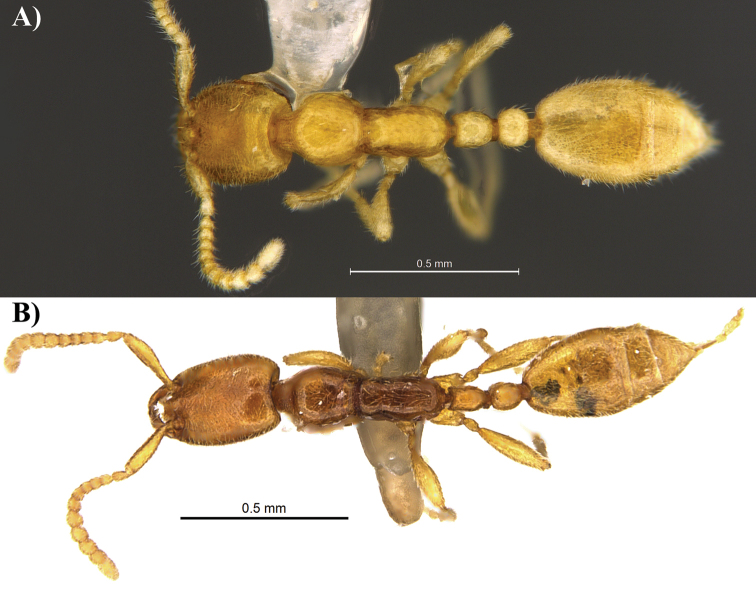
Dorsal view of *Leptanilla clypeata* from Java (**A**) and *Leptanilla hypodracos* from Singapore (Holotype) (**B**). Photographs are on the same scale.

**Figure 5.**
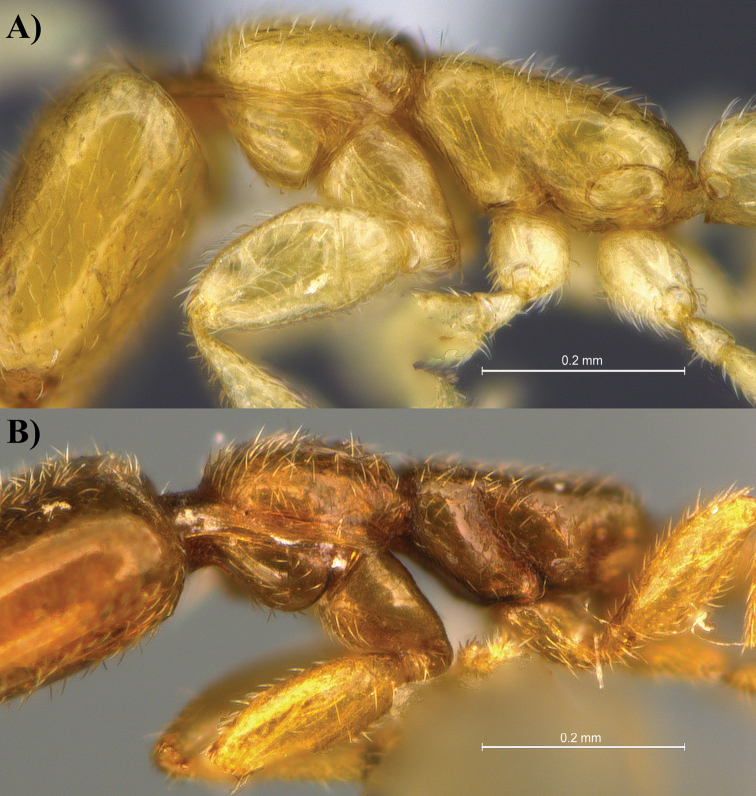
Profile view focusing on the pronotum, mesonotum and anterior part of propodeum of *Leptanilla clypeata* from Java (**A**) and *Leptanilla hypodracos* from Singapore (Holotype) (**B**). Note that the right side of *Leptanilla hypodracos* is presented here. Photographs are on the same scale.

**Figure 6.**
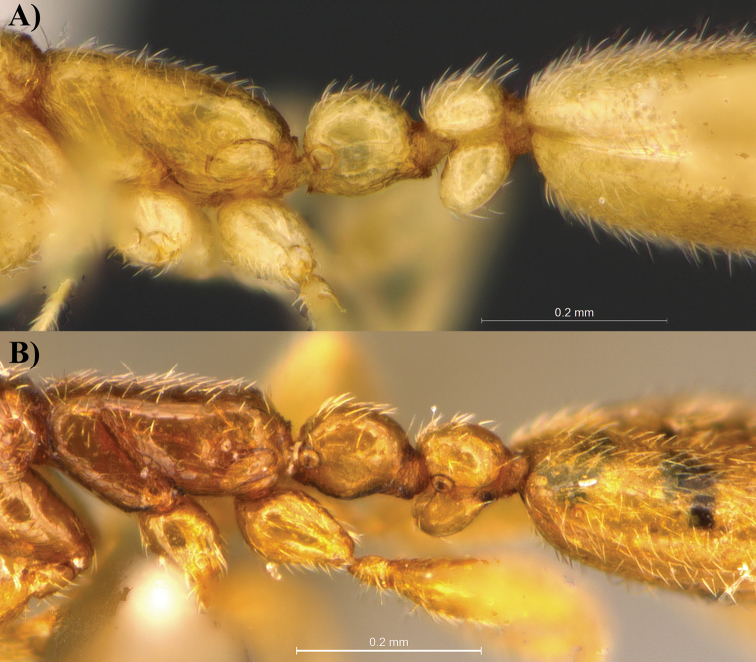
Profile view focusing on the propodeum, petiole and subpetiole of *Leptanilla clypeata* from Java (**A**) and *Leptanilla hypodracos* from Singapore (Holotype) (**B**). Photographs are on the same scale.

**Figure 7.**
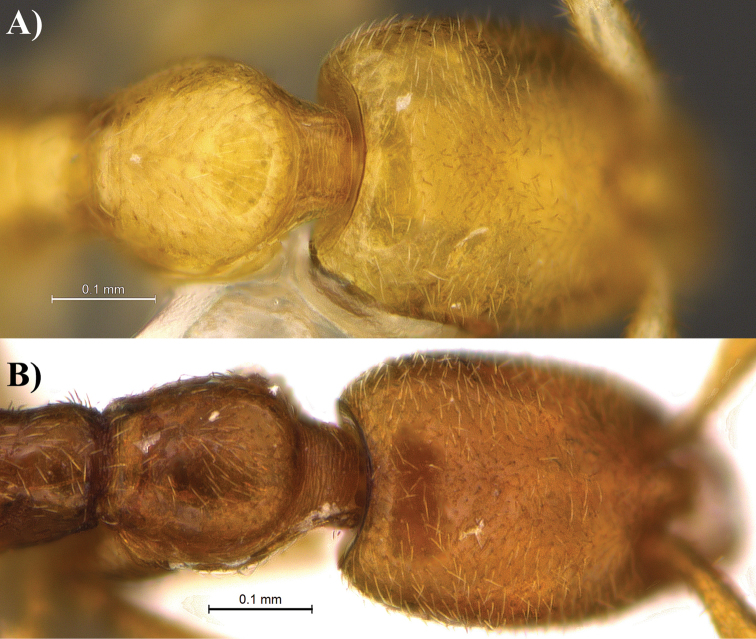
Dorsal view focusing on the pronotum and neck of *Leptanilla clypeata* from Java (**A**) and *Leptanilla hypodracos* from Singapore (Holotype) (**B**). Photographs are on the same scale.

Figs 1 , 2 , 3 , 4 , 5 , 6 , 7

##### Holotype.

Worker from SINGAPORE, Central Catchment Nature Reserve, 1°21.3'N; 103°48.9'E, ca. 55m asl, 15.VI.2015, via subterranean pitfall trap, leg. Mark K. L. Wong, label “MW150615-1.1” deposited in LKCNHM.

##### Paratypes.

Two workers in total, all with the same data as holotype (deposited at SBSHKU), labelled “MW150615-1.2” and “MW150615-1.3”. Unfortunately these specimens were incomplete when collected, with only a head present for one specimen and the second specimen missing part of its antennae and legs. Both specimens were probably damaged by other ants present in the subterranean pitfall trap during collection. The second specimen was very fragile and was damaged during specimen manipulation (breakage at the propodeum/petiole junction). The broken parts were kept in ethanol while the head and mesosoma were mounted for measurements.

##### Measurements and indices.

**Holotype.** HL 0.35 mm; HW 0.27 mm; MaL 0.16 mm; SL 0.19 mm; EL 0 mm (eye absent); WL 0.44 mm; PNW 0.18 mm; PNH 0.12 mm; MW 0.11 mm; PTL 0.10 mm; PTW 0.06 mm; PTH 0.10 mm; TL 1.73 mm (stinger not included); PPL 0.09 mm; PPW 0.08 mm; PPH 0.12 mm; CI 78, SI 69, MaI 57, PI 60, PPI 90, PPHI 70.

**Paratype** (n = 1). HL 0.35 mm; HW 0.27 mm; MaL 0.15 mm; SL 0.19 mm; WL 0.44 mm; PNW 0.18 mm; PNH 0.12 mm; MW 0.11 mm; CI 76, SI 69, MaI 54.

##### Worker description.

**Head.** Head longer than wide (CI = 76–78). In full-face view, posterior margin of head straight to slightly concave. Lateral margins of head slightly convex with posterior margins rounded. Eyes absent. Anterior clypeal margin extending forward with two rounded lobes anterolaterally and slightly concave on its anteromedian portion (Fig. 1B). Median portion of clypeus raised. Mandibles short relative to head (MaI = 54–57) armed with three teeth. Mandibles with a distinct ridge on their basal margin (Fig. 2B). Apical tooth large and acute followed by two smaller teeth; with the basal tooth significantly smaller, blunt and pointing inward. Antennal insertion exposed. Antennae with 12 segments. Scape inflated in its median portion, dorsally concave, and extending over the mid-point of head. Pedicel distinct from the scape and flagellum by marked constrictions. Flagellum incrassate with the last antennal segment distinctly longer than the previous flagellomeres, about the size of the previous two segments.

**Mesosoma.** In lateral view, mesosoma with a continuous straight appearance with the exception of a well-marked interruption of the promesonotal suture (Fig. 3B). In dorsal view, pronotum wider than posterior portions of mesosoma, especially on its anterior half (Fig. 4B). In profile view, a distinct sulcus of fine striae separates the pronotum from the propleuron extending from the dorsal portion of the neck and reaching the inferior part of the promesonotal suture just above the coxal junction (Fig. 5B). In profile view, both anterodorsal and anteroventral parts of pronotum rounded with the latter droplet-shaped. Mesonotum and propodeum with similar width and without obvious inflated portion. Promesonotal suture deeply impressed and clearly visible in both dorsal and profile view. Metapleural gland bulla large and elongate, nearly as large as the maximum width of hind coxa (Fig. 6B). Posterior part of propodeum forming the propodeal declivity nearly at right angle with rounded edges.

**Metasoma.** In profile view, dorsal and ventral portion of petiolar node markedly convex, rounded without acute portion nor subpetiolar process. Dorsal margin of postpetiole convex and rounded. Dorsal margin of the postpetiolar node lower than the maximal height of the dorsal margin of the petiolar node. Sternopostpetiolar process well developed and rounded. In dorsal view, petiolar node longer than wide (PTL = 0.10 mm, PTW = 0.06 mm) while postpetiole more rounded (PPL = 0.09 mm, PPW = 0.08 mm).

**Sculpture.** Sculpture absent on most of the body. Most of the body with a slick and shiny appearance with the exception of the neck with clear transversal striae (Fig. 7B). Hair insertion on head giving an impression of the presence of small punctuations.

**Pubescence.** Pubescence present on most of the body, especially on dorsal parts. Antennae and mandibles with numerous erect to suberect long hairs.

**Coloration.** Head, thorax and fore coxa with a dark amber colour, while petiole, postpetiole and most of the gaster slightly lighter. Antennae, legs (with the exception of the fore coxa) and tip of the gaster with a much lighter yellow coloration.

**Castes.** Male and female unknown.

##### Etymology.

The species epithet is derived from a combination of the Latin terms for ‘under’ and ‘dragon’, in reference to the slender, dragon-like appearance of this subterranean predator. The species epithet is a noun, and thus invariant.

##### Distribution.

Southeast Asia. Only known from Singapore.

##### Ecology.

*Leptanilla hypodracos* was collected from a well-shaded patch of tropical low-lying old secondary forest with a high density of leaf litter and woody debris on the forest floor. As with other Leptanillinae, *Leptanilla hypodracos* presents a hypogaeic lifestyle and was collected in a baited subterranean pitfall trap at a depth between 10–15 cm. Colony size and structure is unknown. Although the specimens were collected in a trap containing tuna bait, it is presently unclear as to whether *Leptanilla hypodracos* were recruited to the bait, since other *Leptanilla* species have previously been suggested to be specialist predators of geophilomorph centipedes (Masuko 1990) and have yet to demonstrate any scavenging habits. In contrast, other species present at the same bait were collected in high densities (>100 individuals each), and these belonged to mass-raiding species of *Dorylus* Fabricius, 1973 and generalized foraging species of *Carebara* Westwood, 1840.

##### Remarks.

Based on a morphological examination, *Leptanilla hypodracos* is close to several other *Leptanilla* from the Oriental region, namely *Leptanilla escheri* Kutter, 1948, *Leptanilla butteli* Forel, 1913, and *Leptanilla thai*, but is most similar to *Leptanilla clypeata*.

*Leptanilla hypodracos* differs from *Leptanilla escheri* in the anterior margin of the petiole in profile view, which is rounded in *Leptanilla hypodracos* but more angular in *Leptanilla escheri* while in dorsal view, *Leptanilla hypodracos* displays a long and narrow petiole (PI = 47, PPI = 83) that contrasts with the rounded petiole which is as wide as it is long in *Leptanilla escheri* (PI = 87–120, PPI = 117–145). In dorsal view *Leptanilla hypodracos* also has a narrower mesosoma than *Leptanilla escheri*. Furthermore, as records of *Leptanilla escheri* are restricted to southern Indian highlands where the elevation ranges from 1250 to 1775m asl, and *Leptanilla hypodracos* was collected in a tropical lowland forest of Singapore at an elevation of 55m asl, it is conceivable that the two species occupy differing ecological niches.

Similar species reported from the Malay Peninsula include *Leptanilla butteli* from West Malaysia and *Leptanilla thai* from Southern Thailand. However, *Leptanilla hypodracos* is distinguished from these species in having a more rounded petiolar node and a less inflated petiole. In comparison, both *Leptanilla butteli* and *Leptanilla thai* possess square-shaped petiolar nodes with rounded angles and more inflated petioles (Baroni Urbani 1977). In full-face view, the extension of the anterior clypeal margin is present in *Leptanilla hypodracos* but absent in *Leptanilla butteli*. In terms of overall size, *Leptanilla hypodracos* (TL = 1.73 mm) is larger than both *Leptanilla butteli* (TL = 1.40–1.50 mm) and *Leptanilla thai* (TL = 1.40–1.50 mm), although the head size of *Leptanilla thai* (HL = 0.36 mm, HW = 0.27 mm) (Baroni Urbani 1977) is comparable to that of *Leptanilla hypodracos* (HL = 0.35 mm, HW = 0.27 mm).

*Leptanilla hypodracos* presents the most similarities with *Leptanilla clypeata* from Java, Indonesia, but can be distinguished from the latter by a suite of distinct characteristics. We also provide a new set of complete measurements for *Leptanilla clypeata* (see below). The primary difference is observed in dorsal view and pertains to the shape and size of the petiolar node and postpetiole. In *Leptanilla hypodracos*, the petiolar node is nearly twice longer than wide (PI = 60, PTL = 0.10 mm, PTW = 0.06 mm) and the postpetiole is longer than wide, and also more rounded (PPI = 90, PPL = 0.9 mm, PPW = 0.08 mm). However in *Leptanilla clypeata*, the petiolar node is almost as wide as long (PI = 82, PTL = 0.11 mm, PTW = 0.09 mm in Ito et al. 2001; new measurements PI = 100, PTL = 0.10 mm, PTW = 0.10 mm), while the postpetiole is distinctly wider than long (PPI = 137, PPW = 0.11 mm, PPL = 0.08 mm in Ito et al. 2001; new measurements PPI = 133, PPW = 0.12 mm, PPL = 0.09 mm) contrary to *Leptanilla hypodracos*. Hairs on the posterodorsal subpetiolar process of *Leptanilla hypodracos* are sparser and shorter than in *Leptanilla clypeata*. The posterodorsal corner of the prodeum also more angular in *Leptanilla hypodracos* while being more rounded in *Leptanilla clypeata*. In full-face view, the mandibles of *Leptanilla hypodracos* display a long and well-defined basal tooth, however this character is neither visible in the observed specimen of *Leptanilla clypeata* nor on the figures of Ito et al. (2001, Figures 7A, C).

In addition to the characteristics above, there are several other differences between *Leptanilla hypodracos* and *Leptanilla clypeata*, which should be confirmed with future collection of both species. The head of *Leptanilla hypodracos* (CI = 76–78) is slightly narrower than that of *Leptanilla clypeata* (CI = 82 in Ito et. al. 2001, and CI = 84 with our measurements below) (Fig. 1). In profile view, the metanotal groove appears more deeply impressed in *Leptanilla hypodracos* than it does in *Leptanilla clypeata*. In full-face view, the anterior part of the clypeal projection is slightly concave to straight in *Leptanilla hypodracos* but deeply concave in *Leptanilla clypeata*. Lastly, in dorsal view the pronotum of *Leptanilla hypodracos* (PNW = 0.18 mm) is narrower than that of *Leptanilla clypeata* (PNW = 0.20 mm).

### Measurements and indices for *Leptanilla clypeata*

The specimen (Figs 1–7) used below for measurement was collected by Fumitori Ito in Kebun Raya Bogor, Java (Indonesia) on December 16^th^ 2000, in the same general area of the holotype and paratypes of *Leptanilla clypeata*, and subsequently lent to the authors for measurements.

**Measurements.** HL 0.37 mm; HW 0.31 mm; MaL 0.16 mm; SL 0.20 mm; WL 0.48 mm; PNW 0.20 mm; PNH 0.14 mm; MW 0.12 mm; PTL 0.10 mm; PTW 0.10 mm; PTH 0.11 mm; TL 1.79 mm (stinger not included), PPL 0.09 mm; PPW 0.12 mm; PPH 0.15 mm; CI 84; SI 65, MaI 53; PI 100; PPI 133, PPHI 80.

### New record of *Leptanilla* from Hong Kong and southeastern China

A single worker of a *Leptanilla* species identified as Leptanilla sp. cf. japonica is reported from Hong Kong. The specimen was collected in leaf litter using the Winkler extractor method in Lung Fu Shan Park (22°16.823'N, 114°8.270'E, 116m), located on Hong Kong Island on November 25^th^ 2014. The specimen is deposited at the California Academy of Sciences, San Francisco, California, U.S.A. under the specimen code CASENT0914941. Photographs of the specimen are available on Antweb.org. While the specimen appears to match the description and characteristics of *Leptanilla japonica*, we are hesitant to formerly identify it as such due to the important disjunction observed in the distribution of this species. To the best of our knowledge this species has been reported only from Honshu Island in Japan (Antmaps 2015) and it is thus surprising to find it so far south (≥2500km) in the absence of other intermediate records. Future surveys in Asia should reveal if this record represents a valid record of *Leptanilla japonica* or a new cryptic species morphologically similar to the latter.

### Synoptic species list of Oriental *Leptanilla* species

*Leptanilla astylina* Petersen, 1968 (Philippines) – described from male only

*Leptanilla besucheti* Baroni Urbani, 1977 (Sri Lanka)

*Leptanilla buddhista* Baroni Urbani, 1977 (Nepal)

*Leptanilla butteli* Forel, 1913 (West Malaysia)

*Leptanilla clypeata* Yamane & Ito, 2001 (Java, Indonesia)

*Leptanilla escheri* Kutter, 1948 (South India)

*Leptanilla havilandi* Forel, 1901 (Singapore)

*Leptanilla hunanensis* Tang, Li & Chen, 1992 (Hubei, Hunan & Yunnan, China)

*Leptanilla hypodracos* sp. n. (Singapore)

Leptanilla sp. cf. japonica (Hong Kong)

*Leptanilla kebunraya* Yamane & Ito, 2001 (Java, Indonesia)

*Leptanilla kunmingensis* Xu & Zhang, 2002 (China)

*Leptanilla lamellata* Bharti & Kumar, 2012 (North India)

*Leptanilla santschii* Wheeler & Wheeler, 1930 (Java, Indonesia) – described from male only

*Leptanilla taiwanensis* Ogata, Terayama & Masuko, 1995 (Taiwan)

*Leptanilla thai* Baroni Urbani, 1977 (Thailand)

*Leptanilla yunnanensis* Xu, 2002 (China)

### Updated key to Oriental *Leptanilla* species

The key to Oriental *Leptanilla* species (Bharti and Kumar 2012) is updated with the modifications of sections 3 and 14 and the inclusion of *Leptanilla hypodracos* in a new section (below) and Leptanilla sp. cf. japonica.

Modified after Bharti and Kumar (2012):

**Table:** 

1	Mandible with 2 teeth	**2**
–	Mandible with 3 teeth or more	**3**
2	Anterolateral lobes of clypeus present, 3rd antennal segment with a distinct basal peduncle, postpetiole large, promesonotal suture narrow (Java)	***Leptanilla kebunraya* Yamane & Ito**
–	Anterolateral lobes of clypeus absent, 3rd antennal segment without distinct basal peduncle, postpetiole relatively small, promesonotal suture wide (W. Malaysia)	***Leptanilla butteli* Forel**
3	Mandible with 3 teeth	**4**
–	Mandible with 4 irregular and with the fourth tooth (preapical) very small and difficult to distinguish (Hong Kong)	**Leptanilla sp. cf. japonica**
4	Metanotal groove present	**5**
–	Metanotal groove absent	**6**
5	In full-face view head approximately rectangular. Clypeus not protruding, anterior margin roundly convex. In profile view dorsum of petiole almost straight. In dorsal view postpetiolar node much wider than petiolar node (S. China)	***Leptanilla hunanensis* Tang, Li & Chen**
–	In full-face view head distinctly narrowed anteriorly. Clypeus protruding, anterior margin concave. In lateral view dorsum of petiole roundly convex. In dorsal view postpetiolar node as wide as petiolar node (S. China)	***Leptanilla kunmingensis* Xu & Zhang**
6	Anterior margin of clypeus more or less straight or weakly to strongly convex	**7**
–	Anterior margin of clypeus medially incised, bilobed	**9**
7	Petiole longer ≥ 0.13 mm (Sri Lanka)	***Leptanilla besucheti* Baroni Urbani**
–	Petiole shorter ≤ 0.10 mm	**8**
8	Clypeus slightly protruding anteriorly and with distinctly convex anterior margin, PPI = 122–138, CI ≥ 82, PI = 111–125 (Nepal)	***Leptanilla buddhista* Baroni Urbani**
–	Clypeus not protruding anteriorly and with straight or weakly convex anterior margin, PPI = 163–171, CI ≤ 81, PI = 138–158 (S. China)	***Leptanilla yunnanensis* Xu**
9	Petiole, postpetiole and gaster covered with short and long hairs	**10**
–	Petiole, postpetiole and gaster covered with either short or long hairs	**11**
10	Petiole with an anteroventral lamellate subpetiolar process, ventral face of lamellate process weakly rounded; subpetiolar process with anteroventral and posteroventral corner obtusely angled and posterior face of subpetiolar process weakly concave; petiolar and postpetiolar spiracle almost equal in diameter, PPHI = 74–76; posterior head margin concave (N. India)	***Leptanilla lamellata* Bharti & Kumar**
–	Petiole with a weak subpetiolar process without lamella; petiolar process convex and anteriorly and posteriorly oblique; petiolar spiracle large with a diameter of almost 2 times the diameter of postpetiolar spiracle, PPHI = 85–86, posterior head margin almost straight or weakly concave (S. India)	***Leptanilla escheri* Kutter**
11	Anterior margin of clypeus almost straight with a low median notch (Taiwan)	***Leptanilla taiwanensis* Ogata, Terayama & Masuko**
–	Anterior margin of clypeus medially prominent with deep incision or anteriorly strongly produced and apically distinctly bilobed	**12**
12	Anterior margin of clypeus medially prominent with a deep incision at its apical margin, SI ≥ 74 (Singapore)	***Leptanilla havilandi* Forel**
–	Anterior margin of clypeus with a broad median notch that makes it seem almost bilobed in dorsal view, SI ≤ 68	**13**
13	Clypeus strongly produced anteriorly and having a distinctly raised platform which is defined posteriorly; anterior portion of head lacking a pair of whitish markings	**14**
–	Clypeus not strongly produced anteriorly and lacking a distinctly raised platform which is defined posteriorly; anterior portion of head with a pair of whitish markings (Thailand)	***Leptanilla thai* Baroni Urbani**
14	In dorsal view petiolar node almost twice as long as wide and postpetiolar node almost as wide as long, PI = 60, PPI = 90 (Fig. 4B); in profile view propodeal declivity is more angular (Fig. 3B). In full-face view, mandibles with a long and well-defined basal tooth (Singapore)	***Leptanilla hypodracos* sp. n.**
–	In dorsal view petiolar node almost as wide as long and postpetiolar node distinctly wider than long, PI = 82–100, PPI = 133–137 (Fig. 4A); in profile view propodeal declivity is more rounded (Fig. 3A). In full-face view, mandibles lacking a long and well-defined basal tooth (Java)	***Leptanilla clypeata* Yamane & Ito**

## Discussion

The discovery of *Leptanilla hypodracos* represents a second *Leptanilla* species from Singapore and a fourth species from the Malay Peninsula. Notably, this is the first known record of a *Leptanilla* species from Singapore in over a century, since Forel’s discovery of *Leptanilla havilandi* in 1901. *Leptanilla* species are often considered to be rare ants as they are infrequently collected owing to a minute body size, hypogaeic life history and potentially small colony size (Masuko 1990 reports 100–200 workers in *Leptanilla japonica* colonies). Nevertheless, several methods of collection have been suggested to be particularly effective at targeting *Leptanilla* species. Lopez et al. (1994) report that by using the *lavage de terre* method with large amounts (ca. 40kg) of soil followed by a Burlese-Tullgren extraction, they were able to collect numerous individuals of *Leptanilla charaonea* Barandica, López, Martínez & Ortuno, 1994 (48 specimens) and *Leptanilla zaballosi* Barandica, López, Martínez & Ortuno, 1994 (388 specimens) from single samples, although they also attribute these results to the specific biotope sampled. Other methods of soil extraction have also proven successful in collecting *Leptanilla*. For example, Belshaw and Bolton (1994) collected 30 workers of *Leptanilla boltoni* Baroni Urbani, 1977 by a Winkler extraction of soil samples from 25 × 25 cm quadrats excavated to a depth of 5 cm. Most recently, we collected a single *Leptanilla* worker via a similar method in Hong Kong, which represents the first record of Leptanillinae in this territory and for southeastern mainland China as previous records of *Leptanilla* species for this country are reported only from Hubei, Hunan, and Yunnan (Antmaps 2015). This new record expands the known distribution of the Leptanillinae subfamily in mainland China by nearly 700 km south east. Although the abovementioned collection methods are certainly likely to be effective for collecting *Leptanilla* and other cryptic hypogaiec ants, such broad-scale approaches employing the passive extraction of individuals from large amounts of debris may be limited in their potential for obtaining further biological and ecological information about the species. To this end, we suggest that the usage of baited subterranean pitfall traps (e.g. Schmidt and Solar 2010) may prove an interesting alternative collection method at the fine scale. For example, from our collection of *Leptanilla hypodracos* using small (50 ml) baited subterranean pitfall traps, we were able to confirm the presence of this species at soil depths between 10–15cm based on the position of entrances to the traps and also identify sympatric ant species within the same microhabitat (i.e. *Dorylus* and *Carebara* species). While there is insufficient evidence at present to suggest any recruitment of *Leptanilla hypodracos* to tuna pieces, increased sampling with subterranean pitfall traps using a variety of bait types (e.g. dead vs. live bait) may contribute to our knowledge on the trophic biology of *Leptanilla* species. Considering the relatively small body size of *Leptanilla* ants, future exclusion studies may also be possible by making adjustments to the size of trap entrances such that they prevent larger species from entering. In light of the current shortage of information surrounding cryptic hypogaeic ants such as *Leptanilla* species, the usage of fine-scale collection methods such as baited subterranean pitfall traps can complement other broad-scale approaches so as to obtain the specific biological and ecological information required to achieve a fuller understanding of their life history.

## Supplementary Material

XML Treatment for Leptanilla hypodracos
